# Evaluating conservation biology texts for bias in biodiversity representation

**DOI:** 10.1371/journal.pone.0234877

**Published:** 2020-07-10

**Authors:** Katherine Stahl, Christopher A. Lepczyk, Rebecca A. Christoffel

**Affiliations:** 1 School of Forestry and Wildlife Sciences, Auburn University, Auburn, AL, United States of America; 2 Christoffel Conservation, Madison, WI, United States of America; Instituto de Pesquisas Ecológicas, BRAZIL

## Abstract

A critical component of textbooks is fair representation of the material they cover. Within conservation biology, fair coverage is particularly important given Earth’s breadth of species and diversity of ecosystems. However, research on species tends to be biased towards certain taxonomic groups and geographic areas and their associated ecosystems, so it is possible that textbooks may exhibit similar biases. Considering the possibility of bias, our goal was to evaluate contemporary conservation biology textbooks to determine if they are representative of Earth’s biodiversity. We found that textbooks did not accurately reflect Earth’s biodiversity. Species, ecosystems, and continents were unevenly represented, few examples mentioned genetic diversity, and examples of negative human influence on the environment outweighed positive examples. However, in terms of aquatic versus terrestrial representation, textbooks presented a representative sample. Our findings suggest that modern conservation biology textbooks are biased in their coverage, which could have important consequences for educating our next generation of scientists and practitioners.

## Introduction

Much research and discussion has occurred regarding taxonomic biases (i.e. research is not proportional to organisms’ frequency in nature; [1]) in scientific research, research publications, conservation funding, biodiversity databases, and conservation actions (e.g., species re-introductions). Such work is important because it identifies current knowledge gaps and can help to guide future research [2]. Previous work on taxonomic bias has highlighted that birds and mammals are over-represented in scientific research [1], even though they account for less than 1% of described species diversity [3]. Such biases leave larger groups of organisms vastly under-studied and poorly understood.

Bias also exists within taxonomic groups. For example, butterflies and moths account for only 15% of insect species but were the subjects of 48% of insect studies [1]. Similarly, while sea turtles account for only 0.1% of global reptile species richness, they were the subjects of 20.8% of reptile studies published in the wildlife research literature in the 1990s and 14.0% of such articles published in the 2000s [4]. In the case of reintroduction projects on bird species there was slight overrepresentation of species within the orders Anseriformes (waterfowl), Falconiformes (raptors), Gruiformes (cranes and rails), and Galliformes (gallinaceous birds; [5]).

Taxonomic bias in conservation funding also favors birds and mammals over less charismatic groups [1]. Furthermore, it has been suggested that the level of conservation research and the extinction risk of taxonomic groups are often inversely related [6]. Conservation projects, such as species reintroductions, focus more on charismatic fauna than other groups, with much higher numbers of vertebrate than invertebrate reintroductions [5]. In fact, species selection for reintroduction is based more on funding and societal support than global conservation status [5]. Recently, an analysis of the Global Biodiversity Information Facility (GBIF) examining taxonomic biases found that some groups were grossly under-represented, with just over a third (35% and 36%, respectively) of the known insect and arachnid species being mentioned at least once in the database [7]. In fact, more than half of all occurrences in the database were for birds [7].

Taxonomic bias also exists in holdings that are available in university libraries. For example, birds and mammals were over-represented in holdings while reptiles and amphibians were under-represented [8]. As Hecnar points out, these biases can lead to an incomplete understanding of the biosphere and misunderstandings pertaining to biological principles and their generalizability. Such biases can also lead to misunderstandings regarding the importance and diversity of various taxa and can limit a student’s ability to learn about less well-represented taxa.

Taxonomic bias is not the only type of bias present in conservation science. Bias also exists among the geographic areas in which conservation research, funding, and actions occur. For example, most (>90%) protected area conservation funding comes from and is spent in the world’s richest countries [9], while areas that hold the greatest biodiversity are also the least financially able to fund research and conservation [10]. The types of systems in which conservation science is conducted may also exhibit bias. Terrestrial systems accounted for 74% of studies retrieved in a publication comparing the way in which ecologists of terrestrial and aquatic systems analyze biodiversity [11].

Understanding whether or not bias exists in conservation biology textbooks is critical, as misrepresentation in literature contributes to the cycle of a species being under-funded, under-studied, and relatively unknown by future generations of conservation scientists. Given the preponderance of taxonomic bias in research and the media, our overarching goal was to evaluate whether or not conservation science is presenting a more balanced representation of taxa in its disciplinary textbooks. Given that conservation research is biased towards certain taxonomic groups, we predicted that conservation biology textbooks would reflect similar biases, such as an overrepresentation of birds and mammals. We also sought to evaluate if conservation science is representing other forms of biodiversity, such as genetic and ecosystem diversity. We were interested in whether the representation of geographic areas and their associated ecosystems was adequately represented, given the uneven coverage of areas in scientific publications, conservation funding, and conservation research.

## Methods

To determine if bias exists in conservation biology textbooks, we searched the literature for all current or recent (past 15 years) textbooks. We only considered general conservation biology textbooks and not region or taxonomic specific books. Based on these criteria we found seven textbooks as follows: *Conservation Biology for All* [12]; *Key Topics in Conservation Biology 2* [13]; *A Primer of Conservation Biology*, 5^th^ edition [14]; *An Introduction to Conservation Biology* [15]; *Fundamentals of Conservation Biology*, 3^rd^ edition [16]; *Conservation Science*, *Balancing the Needs of People and Nature*, 2^nd^ edition [17]; and *Essentials of Conservation Biology*, 6^th^ edition [18]. These seven books were published by both US (n = 2) and UK (n = 5) publishers. Collectively, these seven books are commonly used for introductory undergraduate courses in conservation biology around the world.

Within each textbook, we evaluated the figures, tables, and boxes as examples of the type of diversity presented. To avoid any differences in interpretation, the lead author conducted all the data entry. Specifically, from each figure, table, and box, we recorded taxonomic group (if a specific species or group was described or shown in an image), if the species or taxonomic group was an aquatic or terrestrial organism, the continent where data were gathered, ecosystem type (if specifically stated in the example or if it could be identified), and if the example mentioned genetic diversity (if specifically stated in the example or if it could be inferred). Because there is not a singular source for classifying ecosystems, we grouped them into 15 broad classes that are representative of ecosystem descriptions in the literature and from Bailey [19]. Upon compiling data across the seven texts, we then tabulated overall totals and proportions and textbook specific totals and proportions for analysis. To determine if the representation of taxonomic groups in textbooks was similar to the group’s relative abundance on Earth (according to [3]), we conducted a chi-square analysis. For human influence and genetic diversity, we simply calculated the percentage of examples presenting such information as a comparative dataset for which analysis does not exist. Finally, for ecosystems, we compared frequency of use among classes to determine how often each ecosystem type was used in examples (Table 1). All analyses were conducted in Microsoft Excel with a p ≤ 0.05 considered significant.

**Table 1:** Ecosystems were sorted into terrestrial and aquatic systems, and then sorted further into 15 broad classes within those groups. Ecoregions were sorted into an additional 5 classes.

Terrestrial		Aquatic		Ecoregion	
Wetland	Tropical wetland	Reef	Reef	Tropical	Countries
	Wetland		Kelp forest		Afrotropic
	Floodplain		Mangrove		Neotropic
Forest	Tropical forest	Marine	Marine	Temperate	Countries
	Rain forest	Ocean	Deep sea	Arctic	Nearctic
	Eucalypt forest		Ocean floor		Antarctic
	Coniferous forest	Fresh	Lake/pond/river		Palearctic
	Temperate forest	Marsh	Marsh	Indo-Malay	Indo-Malay
	Woodland			Australasia	Australasia
Grassland	Grassland				
	Prairie				
Savanna	African savanna				
	Eucalypt savanna				
	Humid savanna				
	Savanna				
	Tropical savanna				
Boreal	Boreal forest				
	Tundra/alpine				
Coast	Coastal				
	Dunes				
Anthropogenic	Agricultural				
	Anthropogenic				
Desert	Desert				
Mountains	Mountains				
	Greater Yellowstone				

## Results

**Fig 1:**
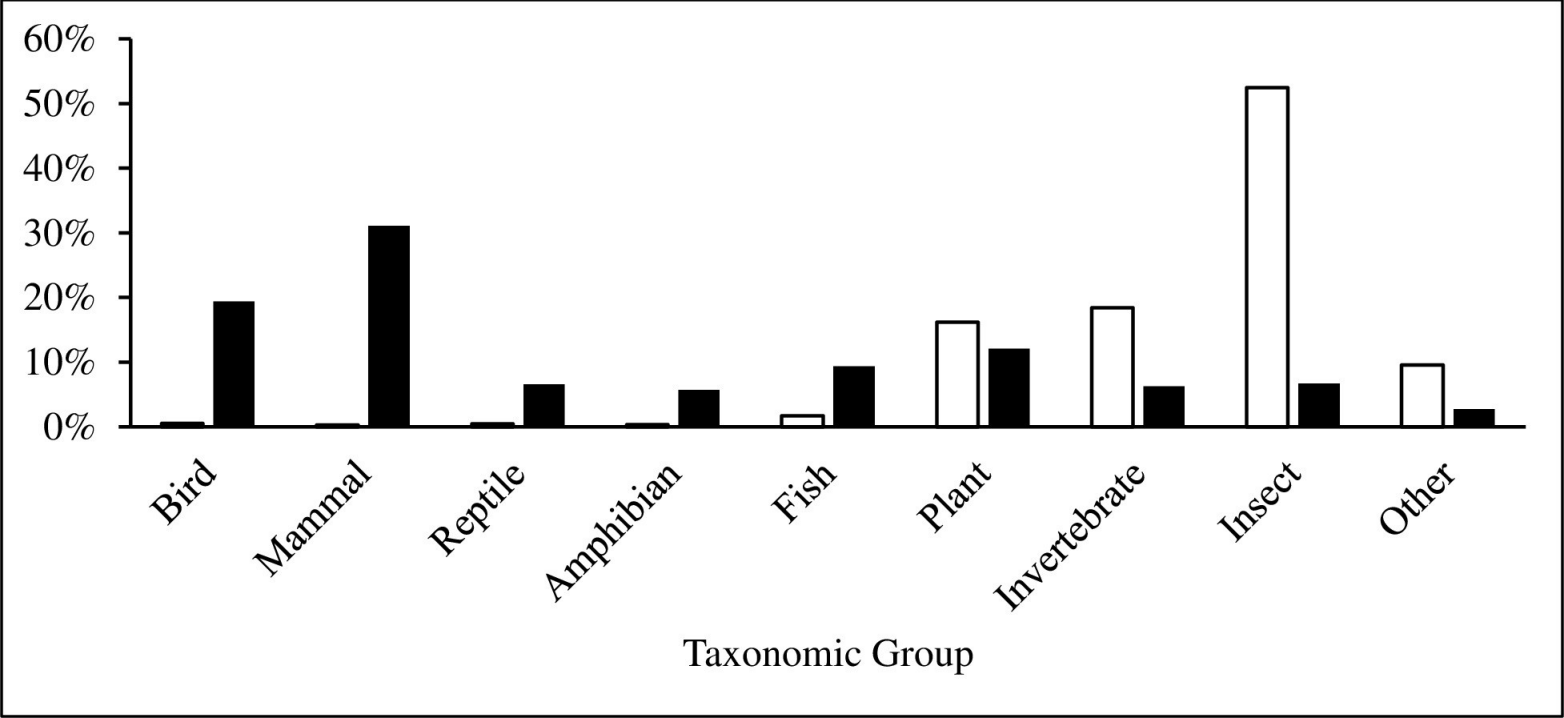
The percentage of conservation textbook examples related to each major taxonomic group. Open bars represent percentages of all taxa represented by each major group. Solid bars represent textbook examples.

**Fig 2:**
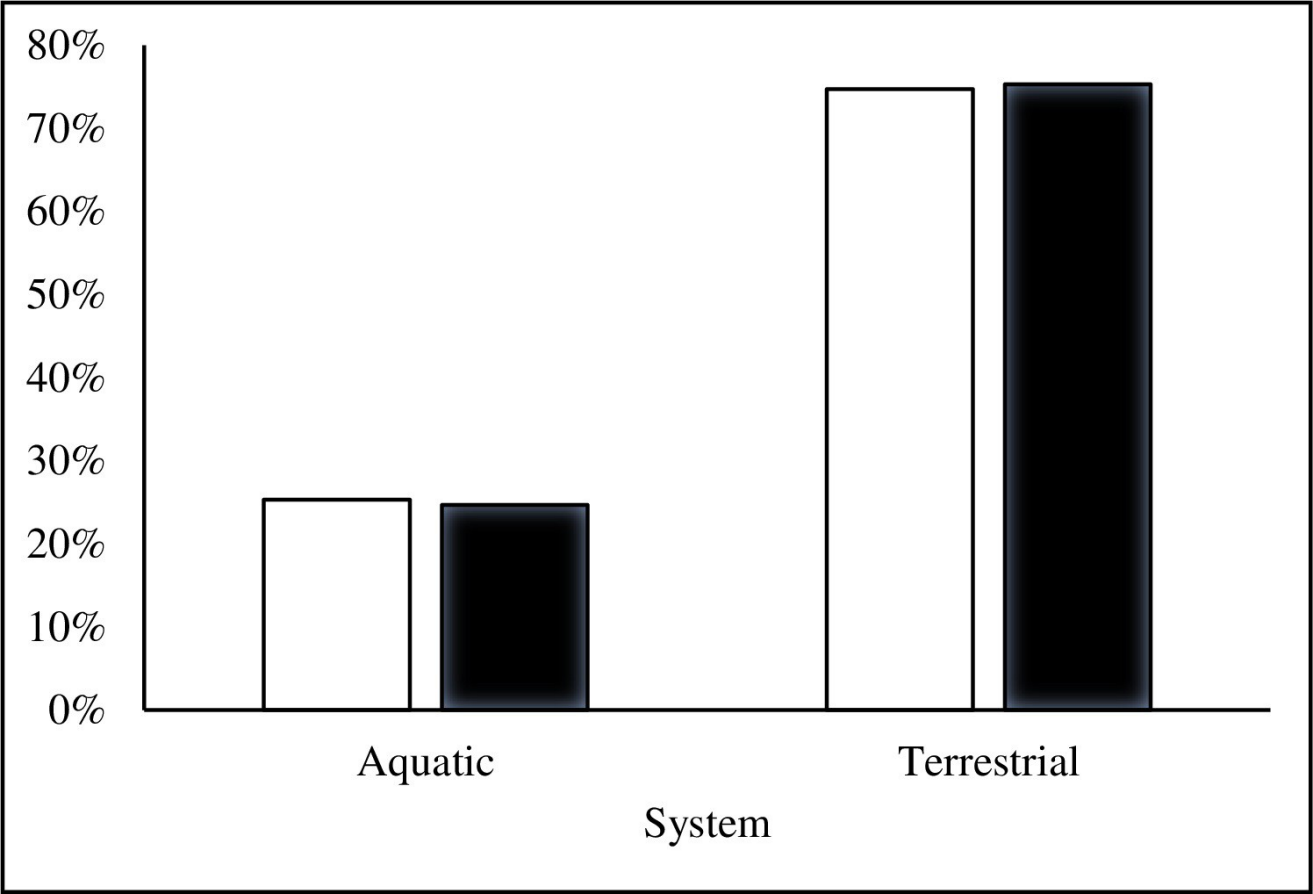
The percentage of conservation textbook examples related to aquatic versus terrestrial systems. Open bars represent percentages of all taxa that are aquatic and terrestrial. Solid bars represent textbook examples.

**Fig 3:**
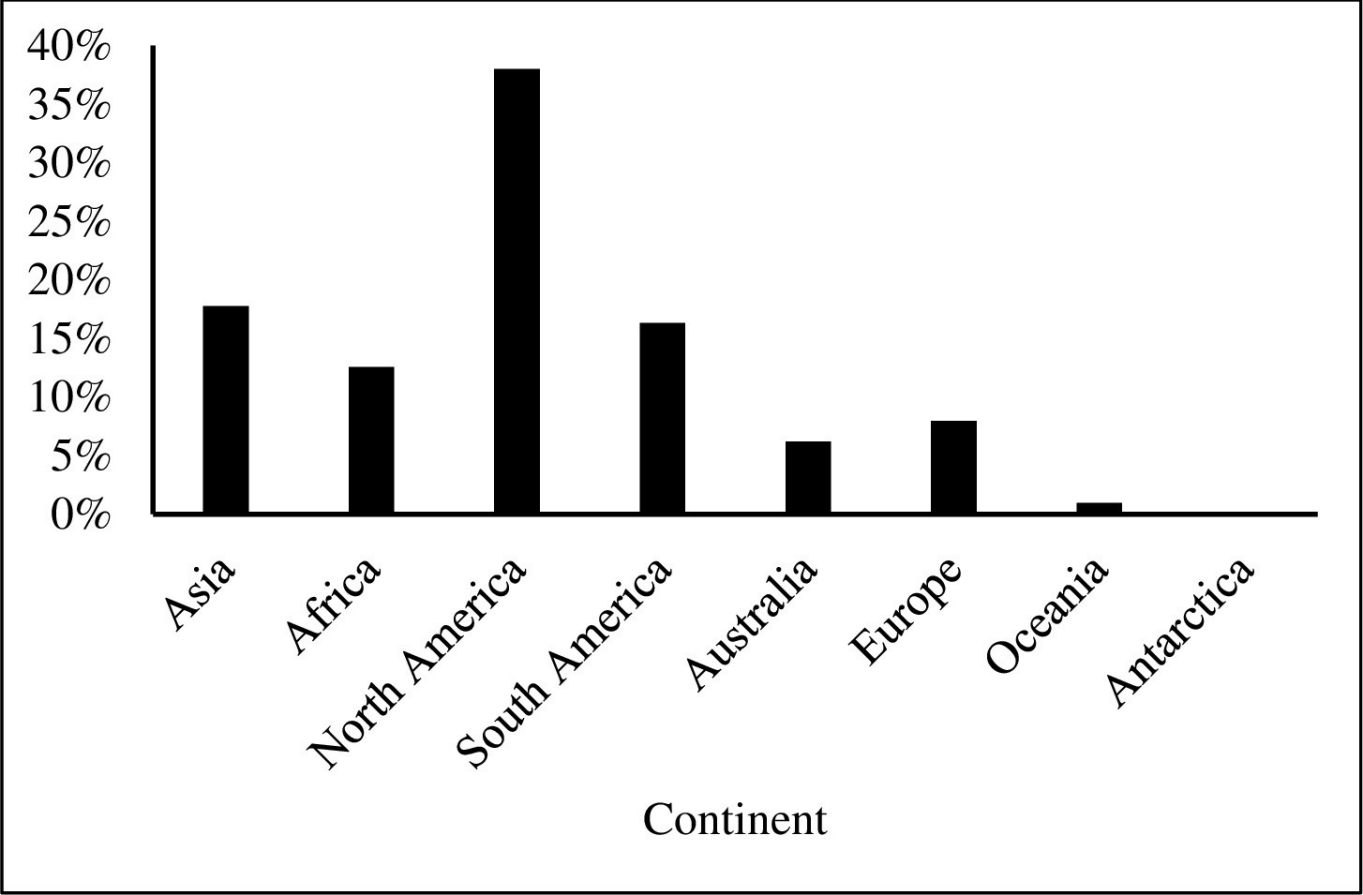
The continent where examples were described as a percent of all examples.

**Fig 4:**
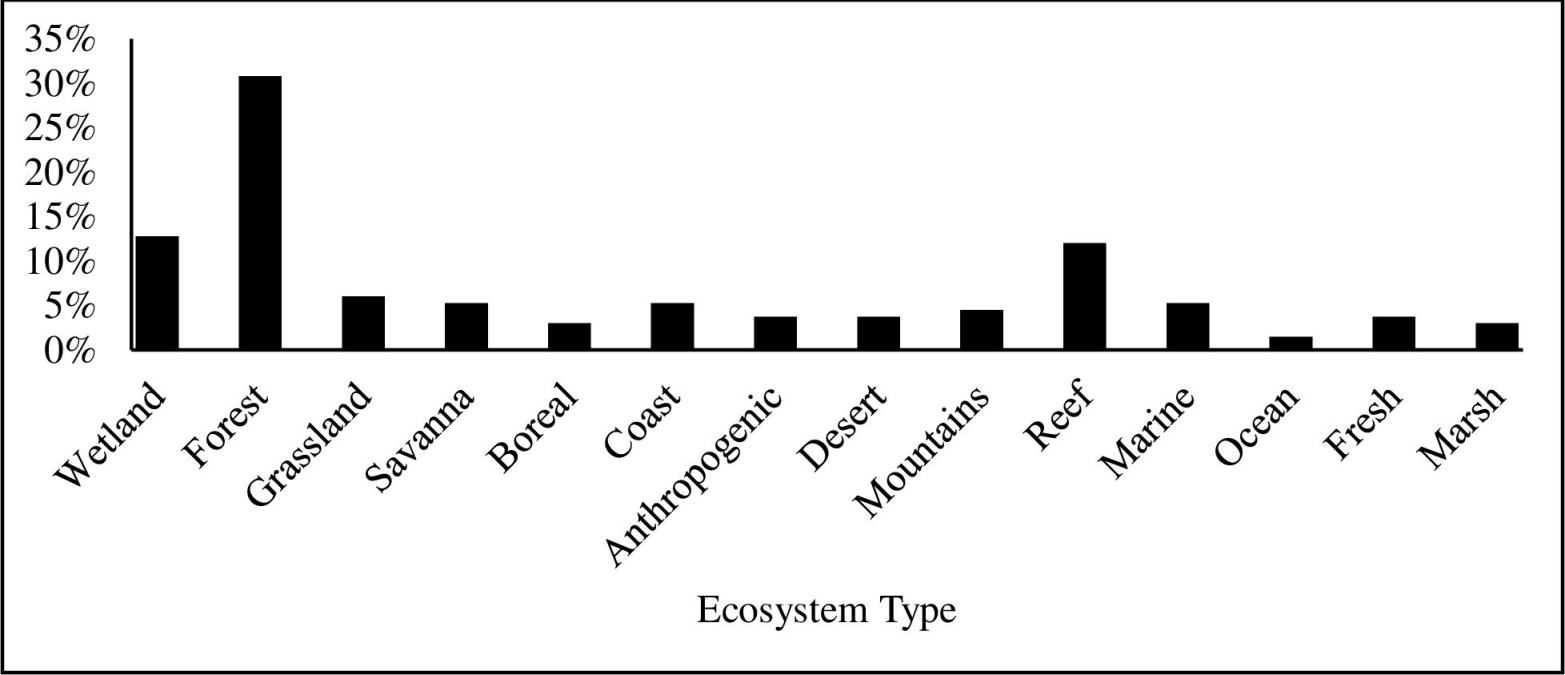
The ecosystem where examples were described as a percent of all examples.

We found that textbooks were significantly biased in their taxonomic coverage of Earth’s biodiversity relative to its actual biodiversity, (χ^2^ = 109.118; df = 8; *p* < 0.001). Of all taxa cited in the textbooks, mammals were used most frequently, accounting for 31% of all examples referring to a specific species or group (Fig 1). Amphibians were the least used example of all main taxonomic groups. Terrestrial organisms were used more frequently as examples than aquatic organisms, but mirrored Earth’s actual aquatic and terrestrial species diversity and hence presented a relatively accurate example of representativeness (Fig 2). In examples where data collection location was mentioned, North America was the most frequently mentioned continent, while Antarctica was the least mentioned site (Fig 3). Only 2.5% of all examples mentioned genetic diversity. Forest ecosystems were the most frequently cited terrestrial ecosystems, while coral reef ecosystems were the most frequently cited aquatic ecosystems (Fig 4). Terrestrial ecosystems were used in more examples (75%) than aquatic ecosystems (25%). In examples mentioning ecoregions, tropical regions were the most frequently mentioned (43%), followed by Arctic (29%) and temperate (14%), while the Indo-Malay (7%) and Australasia (7%) regions were least mentioned. However, only 3 textbooks (*A Primer of Conservation Biology*, 5^th^ edition; *An Introduction to Conservation Biology*; and *Conservation Biology for All*) mentioned ecoregions.

**Fig 5:**
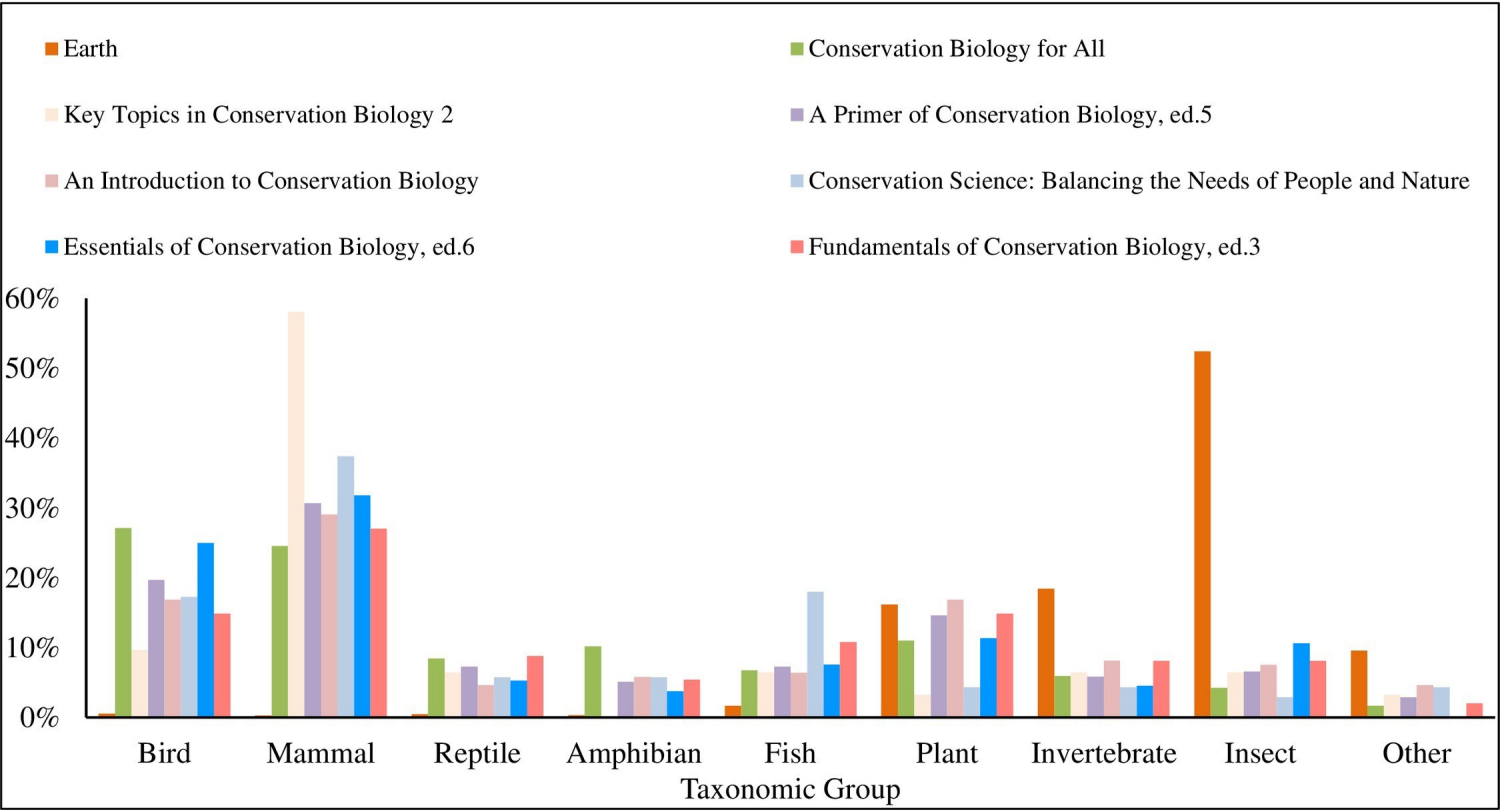
The percentage of conservation textbook examples related to each major taxonomic group as described by each textbook.

**Fig 6:**
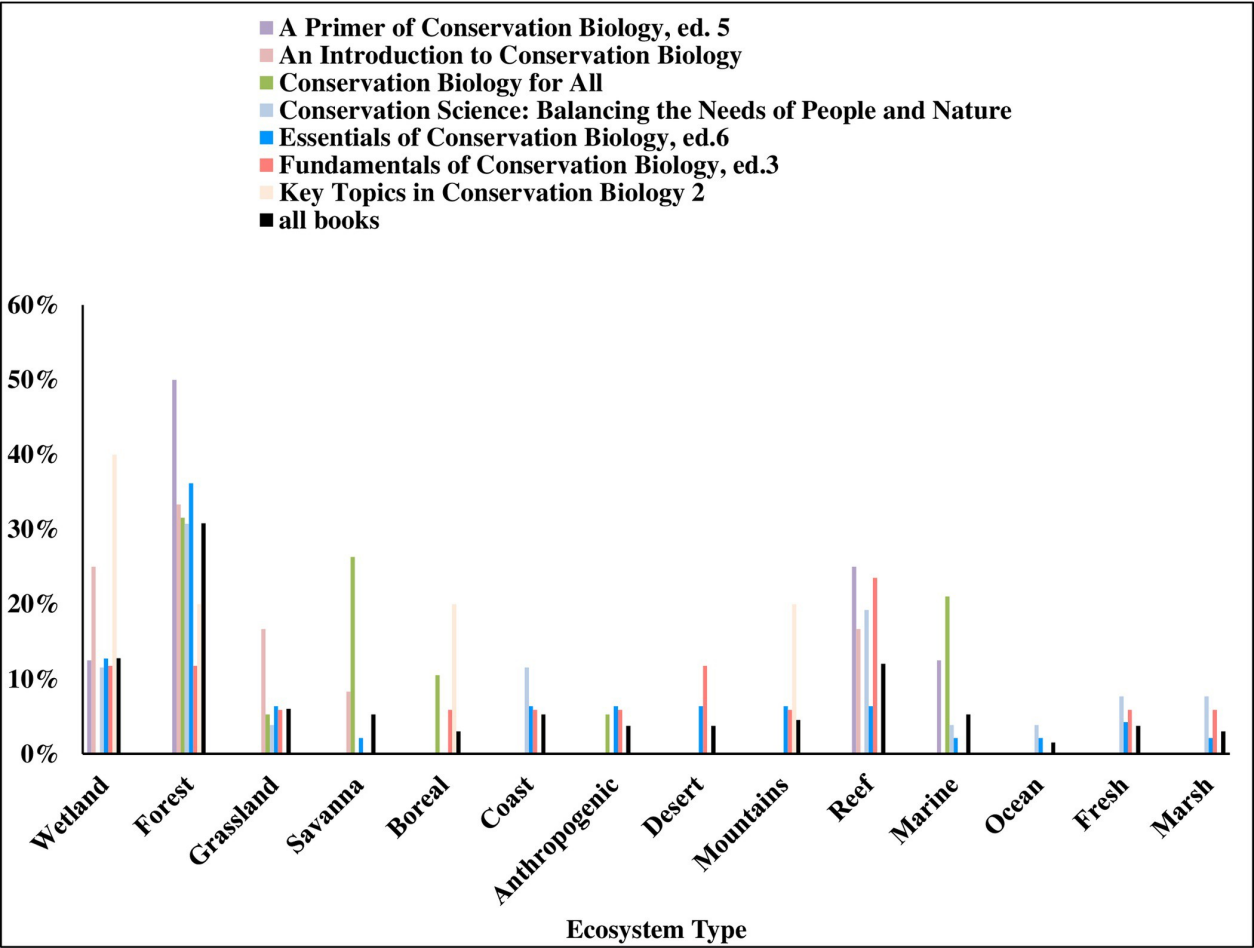
The ecosystem where examples were described as a percent of examples in each textbook.

**Fig 7:**
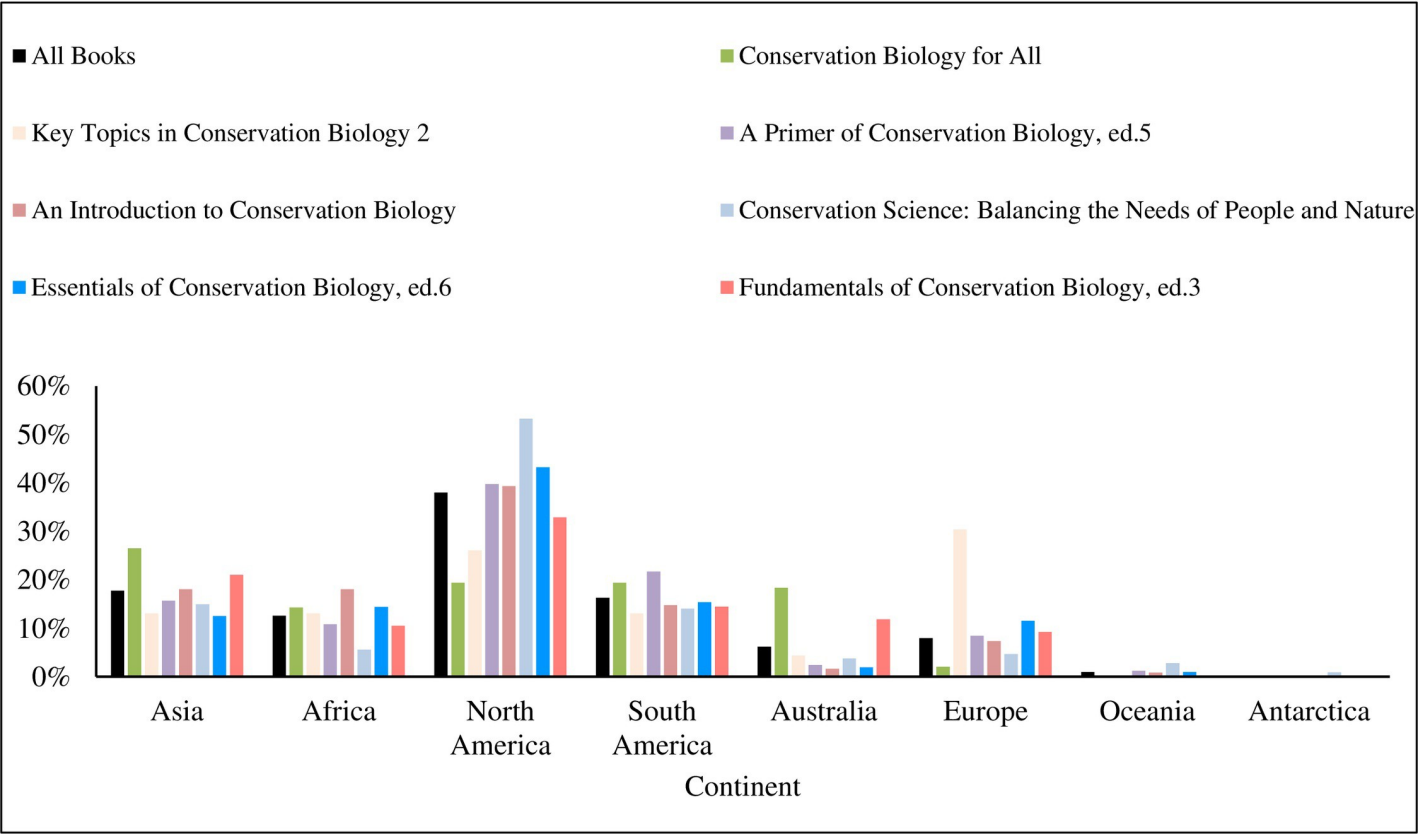
The continent where examples were described as a percent of examples in each textbook.

Within textbooks, *Fundamentals of Conservation Biology*, 3^rd^ edition was the most representative of Earth’s actual biodiversity across taxa, while *Key Topics in Conservation Biology 2* was least representative (Fig 5). *Fundamentals of Conservation Biology*, 3^rd^ edition most frequently mentioned genetic diversity, while *Key Topics in Conservation Biology 2* and *Conservation Biology for All* mentioned genetic diversity the least. Only two textbooks cited an ecosystem other than forest most frequently. *Fundamentals of Conservation Biology*, 3^rd^ edition, mentioned reef ecosystems more than any other type, while *Key Topics in Conservation Biology 2* mentioned wetland ecosystems more than any other type (Fig 6). Similarly, North America was the most common site of data collection in all but two books. *Conservation Biology for All* mentioned Asia as the site of data collection more than any other location, and Europe was the most common data collection site in *Key Topics in Conservation Biology 2* (Fig 7).

## Discussion

Our results supported our prediction that conservation biology textbooks exhibit bias in their coverage of Earth’s biodiversity. Inaccurate representation of taxonomic groups and ecosystems, along with limited examples of genetic diversity, may promote an over-simplified understanding of Earth’s biosphere. All books exhibited some level of bias in their coverage of Earth’s biosphere, especially in their representation of taxonomic groups. *Fundamentals of Conservation Biology*, 3^rd^ edition, most accurately reflected abundances of Earth’s taxa. This book also included more examples of genetic diversity than any other textbook we examined. On the other hand, *Key Topics in Conservation Biology 2* least accurately reflected Earth’s species abundances. Both *Key Topics in Conservation Biology 2* and *Conservation Biology for All* included the fewest examples of genetic diversity. Notably, even though some textbooks were more representative of biodiversity than others, all books exhibited bias. Examples of birds and mammals far outweighed examples of all other taxa, coverage of ecosystems was skewed towards forests, and genetic diversity was rarely discussed.

No other assessments of the biases present in conservation biology textbooks exists. However, Fazey et al. [20] examined the 547 papers published in 2001 in *Biodiversity & Conservation*, *Biological Conservation*, and *Conservation Biology* and found that research reported in these journals was biased toward vertebrates, forests, and natural landscapes. These results are congruent with our findings in terms of the bias toward vertebrates and forests. Fazey et al. [20] also found that community studies and ecosystem studies were under-represented with most studies being of a single species.

The finding that North America was the most frequently mentioned continent (Fig 3) may appear surprising given that the greatest amount of biodiversity lies in the tropics. However, when considering the authors/editors of the textbooks it is important to note that for six of them at least one was from a US institution. More generally, the vast majority (~80%) of ecologists and taxonomists are from North America and Europe [21], which helps to explain the dearth of conservation biology textbooks published in other parts of the world.

The importance of our findings lies in the process of development of conservation scientists through their education, and the call by some authors to address taxonomic biases by having scientists present less charismatic species to the public and develop programs that target these organisms [7]. For students to develop into conservation scientists who are able to present less charismatic species and to develop programs centered on such species, students need to be made aware of these species and be motivated to act [22]. Exposure to these less charismatic species needs to occur within students’ programs of study. For this reason, it is important that educational materials, such as textbooks, accurately represent their topic of interest.

Generally, global hotspots for biodiversity are situated in economically poor countries [10]. This means that conservation funding from non-governmental organizations is critical to protecting threatened and endangered species in those areas [23]. Additionally, rates of extinction in taxonomic groups are directly related to conservation efforts for that group. In terrestrial species, those with large areas of protected habitat experience slower declines than those with small or no areas of protected habitat [24]. Because of this discrepancy wherein protected species benefit more than unprotected species, it is important that conservation efforts target all species of concern, not just charismatic groups. To encourage protection of all at risk taxa, conservation education should offer a broad perspective that extends to all taxonomic groups, ecosystems, and aspects of biodiversity in a more even way. To protect all parts of our world, students need to know and care for all parts of its unique and diverse biosphere–their understanding should not be limited by biases in education materials.

Based on our evaluation of contemporary conservation biology textbooks, we recommend that future textbooks better consider the various ways to measure biodiversity and strive to accurately reflect these measures in their examples (Box 1). We cannot expect students and practitioners to care about species, populations, or ecosystems that they do not understand or have yet to be exposed to. Changing textbooks will require more thought on providing relevant examples and care in describing them, but will potentially result in a more ecologically literate student that has a less biased understanding of the world, and will be motivated and better prepared to share their appreciation and stewardship of less well-known and less-charismatic species.

**Box 1. Suggestions for authors of conservation biology textbooks.**

1. Authors are encouraged to look beyond “conservation science” in their primary literature, and expand to include basic biology and zoology to ferret out diverse examples of taxa used in studies of genetic diversity, etc. For example, think of all the research done using *Drosophila melanogaster* (fruit fly), much of which has focused on genetics of the species.
2. Authors are encouraged to look beyond their existing professional network to include scientists and their work from geographic areas outside of North America and Europe in their textbooks. Such searching might result in future collaborations with individuals from different geographic areas and opportunities to include more examples from such areas, or to co-author future publication of a conservation biology textbook that is more geographically and taxonomically diverse.
3. Authors should encourage students who use their textbooks to look further afield than they themselves have, to explore various taxa and geographic areas, and to pursue funding from non-governmental sources to pursue their passion to study less well-known and less charismatic species. One could include examples from such scientists in their textbooks.
4. Publishers can demand, or at least strongly encourage authors, to include examples from diverse geographic regions, a diverse group of taxa, and/or input from scientists from geographic areas other than North America and Europe.
5. In the case where an author or authors are unable to adequately represent biodiverse examples in their texts, a statement should be included in the beginning of the textbook explaining that the author(s) acknowledge this particular shortcoming in their text and a reason or reasons for this shortcoming.

## Supporting information

S1 Data Evaluating conservation biology texts published data file. (XLSX)
